# Expanded Hemodialysis with Theranova Dialyzer and Residual Kidney Function in Patients Starting Long-Term Hemodialysis: A Randomized Controlled Trial

**DOI:** 10.1681/ASN.0000000655

**Published:** 2025-03-04

**Authors:** Jeong-Hoon Lim, Yu Jin Seo, Yena Jeon, You Hyun Jeon, Hee-Yeon Jung, Ji-Young Choi, Sun-Hee Park, Chan-Duck Kim, Seok Hui Kang, Jung-Hwa Ryu, Duk-Hee Kang, Jang-Hee Cho, Yong-Lim Kim

**Affiliations:** 1 Department of Internal Medicine, School of Medicine, Kyungpook National University, Daegu, Republic of Korea; 2 Department of Statistics, Kyungpook National University, Daegu, Republic of Korea; 3 Department of Epidemiology and Biostatistics, University of California at San Francisco, San Francisco, California; 4 Department of Internal Medicine, Yeungnam University Medical Center, Daegu, Republic of Korea; 5 Division of Nephrology, Department of Internal Medicine, Ewha Womans University School of Medicine, Ewha Medical Research Center, Seoul, Republic of Korea

**Keywords:** dialysis, hemodialysis, ESKD

## Abstract

**Key Points:**

- This randomized controlled trial evaluated the effect of expanded hemodialysis on preserving residual kidney function in patients starting treatment with long-term hemodialysis.
- The expanded hemodialysis group with Theranova dialyzer showed smaller decrease in GFR than the high-flux group over 12 months.
- The Theranova group had a larger reduction in middle molecules and inflammatory cytokines and smaller increases in kidney injury markers.

**Background:**

Expanded hemodialysis using a medium cutoff dialyzer improves the clearance of middle-molecular toxins compared with conventional hemodialysis. This study evaluated the effect of expanded hemodialysis on preserving residual kidney function in patients starting treatment with long-term hemodialysis.

**Methods:**

Patients who initiated long-term hemodialysis were randomized to receive dialysis with either a Theranova 400 (Baxter) or a high-flux dialyzer with a similar surface area over 12 months. The primary outcome was a change in GFR over 12 months, as determined by the mean of urea and creatinine clearance. The secondary outcome was a change in 24-hour urine volume, middle molecules, and kidney injury markers.

**Results:**

A total of 80 patients on hemodialysis (mean age [SD]: 63 [12] years; male: 52 [65%]) underwent randomization. Over 12 months, the Theranova group demonstrated a significantly smaller decrease in GFR than the high-flux group (least squares mean difference of change [95% confidence interval], −1.4 [−2.4 to −0.5] ml/min per 1.73 m^2^). Theranova maintained greater 24-hour urine volume until 9 months, not at 12 months, compared with the high-flux dialyzer. The reduction ratio for κ/λ free light chains, TNF-*α*, and growth differentiation factor-15 was higher in the Theranova group than in the high-flux group. The increase in the kidney injury marker, IGF-binding protein 7, was attenuated in the Theranova group. Hospitalization rate and mortality did not differ between the two groups.

**Conclusions:**

This trial suggests that expanded hemodialysis using the Theranova dialyzer slowed decline in residual kidney function compared with a high-flux dialyzer in patients starting treatment with long-term hemodialysis.

**Clinical Trial registry name and registration number:**

Theranova versus High-flux Dialyzer on Preservation of Residual Renal Function, NCT04211571.

## Visual Abstract

**Figure:**
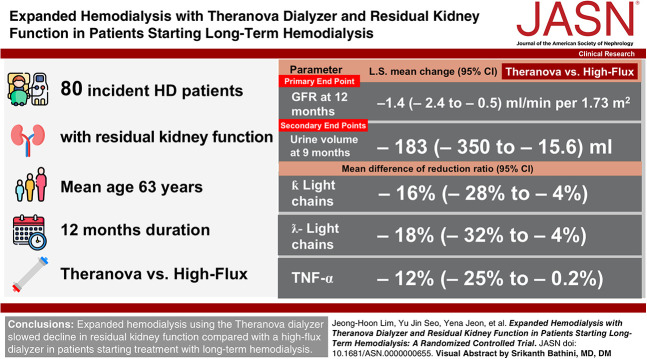

## Introduction

Hemodialysis is the predominant treatment modality for KRT for patients with kidney failure.^1^ However, patients on hemodialysis are vulnerable and have a significantly higher mortality rate than the general population.^2,3^ Therefore, efforts are underway to decrease the mortality rate in these patients. Research in hemodialysis techniques has focused on improving the dialysis membrane to enhance clinical outcomes, including uremic symptoms, complications, and mortality.

Expanded hemodialysis is a dialysis method that uses a medium cutoff dialyzer characterized by enhanced selectivity,^4^ improved permeability,^5^ increased retentive capacity,^6^ and augmented internal filtration.^5^ Expanded hemodialysis selectively increases the clearance of middle-molecular uremic toxins compared with conventional hemodialysis.^7^ Several studies have validated the superior efficacy of expanded hemodialysis over conventional hemodialysis, demonstrating clinical advantages such as improving quality of life and ameliorating pruritus and anemia.^8^

Residual kidney function is consistently associated with clinical outcomes in patients with kidney failure undergoing dialysis, and many studies have verified that residual kidney function is an independent factor associated with mortality.^9–12^ Residual kidney function aids in removing uremic toxins, regulates fluid and electrolyte levels, and manages BP. In addition, residual kidney function helps reduce the burden on the cardiovascular system and improves anemia status by producing erythropoietin.^13,14^ Therefore, preserving residual kidney function in patients with kidney failure is crucial for enhancing quality of life, minimizing complications, and lowering mortality rates.

Most studies on expanded hemodialysis using Theranova have primarily demonstrated the prevention of complications, improvement in subjective symptoms, or removal of middle molecules. However, they did not evaluate the effect of Theranova on residual kidney function in patients on hemodialysis. The aim of this randomized controlled study was to investigate the effect of expanded hemodialysis on preserving residual kidney function during the first year of long-term hemodialysis in patients with kidney failure.

## Methods

### Study Design and Patients

The Theranova versus High-flux Dialyzer on Preservation of Residual Renal Function trial, a multicenter, prospective, randomized, controlled, open-label study, was conducted between April 2020 and September 2023 at four tertiary hospitals in the Republic of Korea (Kyungpook National University Hospital, Kyungpook National University Chilgok Hospital, Yeungnam University Hospital, and Ewha Womans University Seoul Hospital). The protocol was registered in ClinicalTrials.gov (identifier NCT04211571) on December 26, 2019. This study was approved by the institutional review boards of the Kyungpook National University Hospital (KNUH 2020-01-042), Kyungpook National University Chilgok Hospital (KNUCH 2020-07-005), Yeungnam University Hospital (YUMC 2020-07-053), and Ewha Womans University Seoul Hospital (SEUMC 2020-11-009). Written informed consent, where applicable, was obtained from all patients or their legally authorized representatives. This study was conducted in accordance with the tenets of the 2013 Declaration of Helsinki and adhered to the Consolidated Standards of Reporting Trials reporting guidelines.

The study included patients older than 18 years who were newly diagnosed with kidney failure and initiated hemodialysis and met the following criteria: (*1*) hemodialysis for <1 month, (*2*) residual kidney function with 24-hour urine creatinine clearance of more than 2 ml/min, and (*3*) vascular access by arteriovenous fistula/graft or central venous catheter. Patients were excluded if they were scheduled for kidney transplantation within 6 months or had severe hypervolemia state, any hematologic malignancy, monoclonal gammopathy, malignancy, active infectious disease, or HIV infection.

Enrolled patients were randomized to receive treatment with either Theranova 400 (Baxter) or FX CorDiax 80 (Fresenius Medical Care) in a 1:1 ratio using a random number table method with a random number table provided by a statistician who was not involved in the study. Patients and physicians were not blinded to the allocation. All patients received the same treatment from the assigned nephrologist throughout the study period of 12 months. The dialysis time was 4 hours per session with three sessions per week, dialysate flow was 500 ml/min, and blood flow was 200–300 ml/min. Hemoglobin was maintained between 10 and 11 g/dl and phosphate between 2.5 and 4.5 mg/dl. Residual kidney function and serologic tests were followed up every 3 months. The data were collected by the individual trial centers. An interim analysis was not planned for this study.

### Outcomes

The primary outcome was the change in residual kidney function between baseline and 12 months, which was estimated by GFR using 24-hour urine collection. GFR was calculated as the mean of 24-hour urine creatinine and urea clearance, corrected for a body surface area of 1.73 m^2^.

The secondary outcomes were the efficacy and safety of each dialysis treatment. The efficacy outcomes were (*1*) a change in residual kidney function and urine volume every 3 months; (*2*) reduction ratio of middle molecules, such as κ and λ free light chains (FLCs), and inflammatory cytokines, such as TNF-*α* and growth differentiation factor-15 (GDF-15), at 12 months; and (*3*) the change between baseline and 12 months in middle molecules (κ and λ FLCs and *β*2-microglobulin), inflammation-related markers (TNF-*α*, GDF-15, and high-sensitivity C-reactive protein), and kidney injury markers (neutrophil gelatinase-associated lipocalin [NGAL], kidney injury molecule-1 [KIM-1], IGF-binding protein 7 [IGFBP7], tissue inhibitor of metalloproteinases-2 [TIMP-2], and cystatin-C). This article does not report findings with respect to patient-reported outcomes.

### Blood Sampling and Calculation of the Reduction Ratio

Blood samples were collected before and after the midweek dialysis sessions at baseline and after 12 months of dialysis. Postdialysis samples were collected using the slow-flow method,^15^ which involved stopping the dialysate flow, setting the ultrafiltration rate to 50 ml/h, reducing the blood flow rate to 50–100 ml/min for 15 seconds, and then drawing blood samples. The samples were collected in tubes containing a serum-separating agent and then centrifuged for 10 minutes at 3000 rpm and 4°C. Serum samples were immediately frozen and stored at −80°C until analysis. The concentrations of middle molecules, inflammatory cytokines, and kidney injury markers were measured using commercially available ELISA kits: *β*2-microglobulin (molecular weight [MW], 11.8 kDa) with the *β*-2 Microglobulin Human SimpleStep ELISA kit (Abcam, Cambridge, United Kingdom), κ FLCs (monomeric MW, 22.5 kDa) and λ FLCs (dimeric MW, 45.0 kDa) with the Human Ig FLCs Kappa and Lambda ELISA kit (BioVendor-Laboratorni medicina a.s., Brno, Czech Republic), TNF-*α* (MW, 17.4 kDa) with the Human TNF-*α* Quantikine ELISA kit (R&D systems, Minneapolis, MN), GDF-15 (MW, 34 kDa) with the Human GDF-15 Quantikine ELISA kit (R&D systems), KIM-1 (MW, 39 kDa) with the Human Serum TIM-1/KIM-1/hepatitis A virus cellular receptor Quantikine ELISA kit (R&D systems), NGAL (MW, 22 kDa) with the Human Lipocalin-2/NGAL Quantikine ELISA kit (R&D systems), IGFBP7 (MW, 29.1 kDa) with the Human IGFBP7 ELISA kit (Abcam, Cambridge, United Kingdom), and TIMP-2 (MW, 24.3 kDa) with the Human TIMP-2 ELISA kit (Abcam). All assays were performed according to the manufacturer's instructions.

The reduction ratios for each middle molecule and inflammatory cytokine were calculated from the pre- and postdialysis serum concentrations of the corresponding molecules. The reduction ratios of middle molecules were calculated using the Bergstrom and Wehle formula to compensate for hemoconcentration during hemodialysis.^16^ Details of the calculation are shown in our previous study.^8^

### Statistical Analyses

The sample size was determined by the primary outcome. There are no previous studies on the effect of the Theranova dialyzer on residual kidney function. Therefore, we determined the sample size on the basis of a study that compared residual kidney function by dialysis fluid quality. The mean GFR (SD) measured by 24-hour urine collection was 4.3 (1.8) ml/min at 12 months in patients newly initiated on hemodialysis, with a statistically significant difference of more than 1.7 ml/min.^17^ Given this, assuming 80% power, a two-sided type 1 error rate of 5%, and a SD of the residual kidney function difference of 2.5 ml/min, the number of participants needed was 34 in each arm. We also predicted a 15% dropout rate for both groups; thus, a total sample size of 80 was calculated. Statistical analyses were primarily performed by intention to treat (ITT), and patients with at least one measurement of residual kidney function during the study were included in the ITT group.

All variables were expressed as mean (SD) or number (%), depending on the nature and distribution of the variable. Sensitivity analyses were conducted using analysis of covariance (ANCOVA), with the 12-month values set as the dependent variable and the baseline value treated as a covariate. This approach was applied to account for potential differences in baseline measures of the primary and secondary outcomes. *Post hoc* analysis was performed to evaluate the interaction between the treatment effect (dialyzer type) and time whether the change in GFR over the 12 months differed significantly between the groups. This *post hoc* analysis was performed to support the primary outcome and to demonstrate that the trajectory of GFR change over time differed significantly between the Theranova and high-flux groups. For binary outcomes, such as hospitalization and mortality, both risk differences (95% confidence interval [CI]) and relative risk (95% CI) were presented. The least squares (LS) mean and two-sided 95% CI of changes in the GFR, 24-hour urine volume, creatinine clearance, and urea clearance from baseline to the time of assessment in each group were calculated using a constrained longitudinal data analysis model.^18^ This method represents an additional sensitivity analysis aimed at enhancing the validity of the findings and evaluating the long-term effect of different dialyzer types on the preservation of residual kidney function. Statistical analyses were conducted using SPSS version 22.0 (IBM Corp., Armonk, NY) and R (R Foundation for Statistical Computing, Vienna, Austria; www.r-project.org).

## Results

### Patient Population

During the study period, 80 patients on newly initiated hemodialysis were randomized, with 40 patients assigned to each group and included in the ITT analysis. The patients' characteristics, including demographic features, comorbid diseases (diabetes, hypertension, and cardiovascular disease), and laboratory values (hemoglobin, sodium, and potassium) were well balanced at baseline (Table 1). The mean age (SD) was 63 (12) years, and 52 (65%) were men. In both groups, the leading cause of kidney failure was diabetes, followed by hypertension and GN. The antihypertensive and diuretic medications used by both groups during the study are listed in Supplemental Table 1.

**Table 1:** Baseline characteristics

Characteristic	Theranova (*n*=40)	High-Flux (*n*=40)
Age, yr, mean (SD)	63 (11)	62 (13)
Sex, male, No. (%)	28 (70)	24 (60)
Body mass index, kg/m^2^, mean (SD)	23.4 (4.2)	23.4 (3.4)
Body weight, kg, mean (SD)	63 (12)	63 (13)
**Primary kidney disease, No. (%)**		
Diabetes	22 (55)	24 (60)
Hypertension	9 (23)	8 (20)
GN	5 (13)	6 (15)
Others	4 (10)	2 (5)
**Comorbid conditions, No. (%)**		
Diabetes	28 (70)	27 (68)
Hypertension	38 (95)	38 (95)
Cardiovascular disease	6 (15)	8 (20)
Cerebrovascular accident	5 (13)	4 (10)
Predialysis systolic BP, mm Hg, mean (SD)	150 (20)	147 (19)
Predialysis diastolic BP, mm Hg, mean (SD)	69 (11)	70 (13)
Drop out, No. (%)	4 (10)	6 (15)
**Laboratory values, mean (SD)**		
Hemoglobin, g/dl	9.4 (1.3)	9.4 (1.4)
Sodium, mmol/L	138 (4)	137 (4)
Potassium, mmol/L	4.2 (0.7)	4.3 (0.8)
Calcium, mg/dl	8.3 (0.6)	8.2 (0.6)
Phosphate, mg/dl	4.5 (1.3)	4.4 (1.3)
BUN, mg/dl	55 (21)	56 (26)
Creatinine, mg/dl	7.1 (2.4)	6.9 (1.9)
Total cholesterol, mg/dl	136 (36)	146 (35)

**Figure 1:**
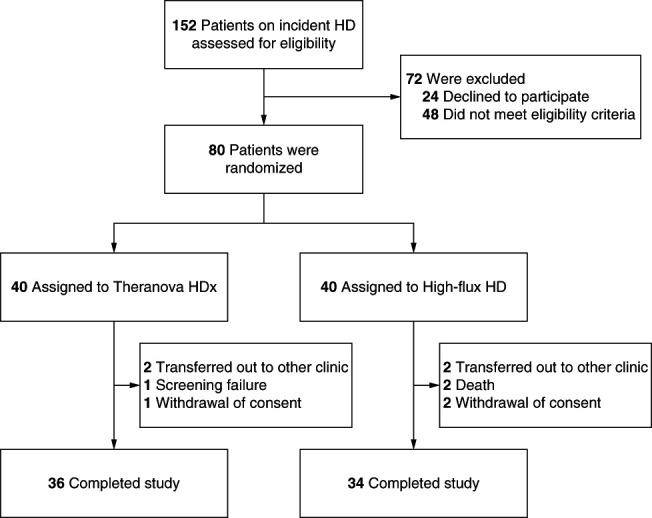
**Flow chart of the THREAD study.** HD, hemodialysis; THREAD, Theranova versus High-flux Dialyzer on Preservation of Residual Renal Function.

Thirty-six patients from the Theranova group and 34 from the high-flux group completed the study and were included in the per-protocol analysis. All analyses were performed on patient groups (as originally assigned). The flow chart of the study (Figure 1) presents the reasons for patient dropouts. The dialysis treatment parameters, including blood flow rate, dialysate flow rate, ultrafiltration volume, and treatment time, did not differ between the two groups (Supplemental Table 2).

### Primary Outcome: Changes in GFR

**Figure 2:**
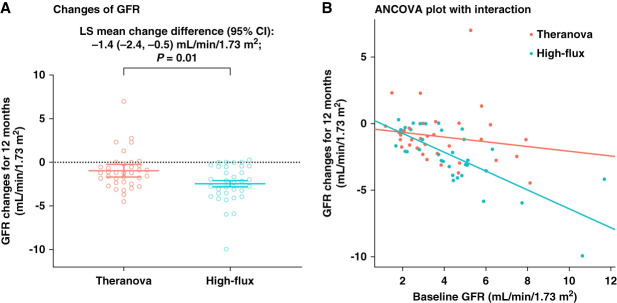
**Changes in GFR from baseline to 12 months.** (A) Individual GFR changes of the study cohort. LS mean difference (95% CI) of GFR change was calculated by an ANCOVA model. Lines indicate mean with 95% CI. (B) *Post hoc* ANCOVA plot with interaction showing the effect of dialyzer type on GFR change between the two groups. ANCOVA, analysis of covariance; CI, confidence interval; LS, least squares.

After 12 months of hemodialysis, both groups experienced a decrease in GFR. However, LS mean (SD) decline in GFR from baseline to 12 months was −1.0 (0.4) ml/min per 1.73 m^2^ in the Theranova group and −2.4 (0.4) ml/min per 1.73 m^2^ in the high-flux group (Table 2). The LS mean difference (95% CI) in change of GFR between the two groups, as determined by ANCOVA adjusted for baseline GFR, was −1.4 (−2.4 to −0.5) ml/min per 1.73 m^2^, significantly favoring the Theranova group than the high-flux group (Figure 2A and Table 2).

**Table 2:** Primary and secondary outcomes in the intention-to-treat population

Parameter	Baseline			12 mo			Changes from Baseline to 12 mo		
	Theranova	High-Flux	Differences (95% CI)	Theranova	High-Flux	Differences (95% CI)	Theranova	High-Flux	LS Mean Differences^a^ (95% CI)
**Primary outcome**									
GFR, ml/min per 1.73 m^2^, mean (SD)	3.7 (1.9)	4.3 (2.2)	0.6 (−0.3 to 1.5)	2.8 (2.5)	2.0 (1.6)	−0.8 (−1.8 to 0.2)	−1.0 (0.4)	−2.4 (0.4)	−1.4 (−2.4 to −0.5)^b^^,^^c^^,^^d^
**Secondary outcomes**									
Efficacy									
*Urine volume, ml/d, mean (SD)*	920.8 (506.7)	927.1 (411.8)	6.3 (−199.3 to 212.0)	704.7 (482.9)	522.4 (375.1)	−182.3 (−388.1 to 23.4)	−230.0 (76.3)	−417.3 (78.5)	−187.3 (−401.8 to 27.3)^b^^,^^c^^,^^d^
*β2-microglobulin, μg/ml, mean (SD)*	18.5 (6.7)	16.7 6 (5.6)	−1.8 (−4.6 to 1.0)	17.6 (4.5)	19.7 (6.3)	2.1 (−0.5 to 4.7)	−0.9 (1.0)	3.0 (1.0)	3.9 (1.2 to 6.6)^b^^,^^c^
*κ FLCs, mg/L, mean (SD)*	47.0 (23.4)	41.5 (24.0)	−5.5 (−16.2 to 5.3)	45.0 (20.6)	42.9 (21.9)	−2.1 (−12.1 to 8.0)	−2.0 (3.7)	1.5 (3.9)	3.4 (−7.0 to 13.8)^c^
*λ FLCs, mg/L, mean (SD)*	70.7 (25.2)	64.2 (28.6)	−6.5 (−18.8 to 5.7)	56.4 (24.7)	51.6 (21.9)	−4.9 (−15.9 to 6.2)	−14.3 (4.2)	−12.3 (34.4)	1.6 (−10.2 to 13.4)^c^^,^^d^
*hs-CRP, mg/dl, mean (SD)*	0.7 (1.2)	0.7 (1.2)	−0.0 (−0.6 to 0.5)	0.2 (0.3)	0.5 (0.6)	0.2 (0.0 to 0.4)	−0.5 (0.2)	−0.2 (0.2)	0.3 (−0.2 to 0.7)
*TNF-α, pg/ml, mean (SD)*	19.2 (8.0)	18.7 (13.1)	−0.6 (−5.5 to 4.4)	16.3 (5.3)	27.0 (68.7)	10.7 (−13.4 to 34.7)	−2.9 (5.6)	8.3 (5.8)	11.3 (−4.5 to 27.0)
*GDF-15, pg/ml, mean (SD)*	8074.6 (2237.8)	7696.8 (2324.4)	−377.8 (−1414.1 to −658.5)	9074.7 (2879.3)	9070.1 (2499.8)	−4.6 (−1278.7 to 1269.6)	1000.1 (409.8)	1373.3 (427.5)	373.2 (−787.5 to 1534.0)^c^
*KIM-1, pg/ml, mean (SD)*	496.6 (754.0)	302.7 (234.3)	−193.9 (−448.8 to 60.9)	496.1 (506.0)	492.9 (356.0)	−3.2 (−209.3 to 202.9)	−0.5 (83.4)	190.2 (87.0)	190.8 (−45.4 to 426.9)^c^^,^^d^
*NGAL, ng/ml, mean (SD)*	13.7 (11.4)	13.3 (11.9)	−0.3 (−5.6 to 5.0)	13.6 (9.7)	15.8 (10.9)	2.2 (−2.7 to 7.1)	−0.1 (1.8)	2.5 (1.9)	2.5 (−2.6 to 7.7)
*IGFBP7, ng/ml, mean (SD)*	38.1 (17.6)	41.0 (29.0)	2.9 (−8.1 to 13.9)	43.9 (19.4)	56.8 (24.2)	12.9 (2.4 to 23.4)^b^	5.9 (3.8)	15.8 (3.9)	10.0 (−0.7 to 20.7)^b^^,^^c^^,^^d^
*TIMP-2, pg/ml, mean (SD)*	4416.7 (1159.9)	4646.6 (1587.4)	229.9 (−403.7 to 863.5)	4746.6 (1385.3)	4518.1 (937.6)	−228.5 (−785.5 to 328.6)	329.9 (213.3)	−128.5 (222.5)	−458.4 (−1062.6 to 145.8)
*Cystatin-C, mg/l, mean (SD)*	5.1 (1.0)	4.9 (0.7)	−0.1 (−0.5 to 0.3)	5.7 (1.1)	5.7 (1.0)	0.0 (−0.5 to 0.6)	0.6 (0.2)	0.8 (0.2)	0.2 (−0.3 to 0.6)^c^
Safety, No. (%)						Risk difference (95% CI); relative risk (95% CI)			
*Hospitalization*	NA	NA		6 (15)	5 (13)	−2.5 (−17.6 to 12.6); 1.13 (0.38 to 3.37)	NA	NA	
*Mortality*	NA	NA		0	2 (5)	5.0 (−1.8 to 11.8); 0.18 (0.01 to 3.80)	NA	NA	

CI, confidence interval; FLC, free light chain; GDF-15, growth differentiation factor-15; hs-CRP, high-sensitivity C-reactive protein; IGFBP7, IGF-binding protein 7; KIM-1, kidney injury molecule-1; LS, least squares; NA, not applicable; NGAL, neutrophil gelatinase–associated lipocalin; TIMP-2, tissue inhibitor of metalloproteinases 2.

a The differences in the primary and secondary efficacy variables between groups were calculated using an analysis of covariance model adjusted for baseline measurements of each variable.

b Group effect is significant (*P* < 0.05).

c Time effect is significant (*P* < 0.05).

d Group and time interaction effect is significant (*P* < 0.05).

To investigate further the effect of dialyzer type on GFR changes, a *post hoc* ANCOVA analysis was performed to evaluate the interaction between group and time. This analysis was performed to determine whether the patterns of GFR change over 12 months differed significantly between the Theranova and high-flux groups. The results showed a significant group×time interaction (*P* = 0.01), indicating that the trajectory of GFR changes over the 12-month period differed between the two groups (Figure 2B). Specifically, patients in the Theranova group had a smaller decline in GFR compared with those in the high-flux group, regardless of baseline GFR. This *post hoc* analysis provides additional support for the primary outcome.

### Secondary Efficacy Outcomes

#### GFR

**Figure 3:**
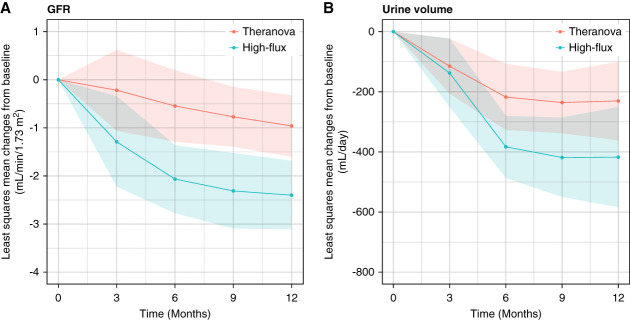
**Changes in GFR and urine volume over the study period.** LS mean changes from baseline to 12 months for GFR (A) and 24-hour urine volume (B). Data are presented as LS mean changes with 95% CIs, which were calculated using a constrained longitudinal data analysis model.

The LS mean changes (95% CI) of GFR from baseline to 6, 9, and 12 months were consistently smaller in the Theranova group compared with the high-flux group (6 months: −1.5 [−2.6 to −0.5] ml/min per 1.73 m^2^; 9 months: −1.5 [−2.6 to −0.5] ml/min per 1.73 m^2^; 12 months: −1.4 [−2.4 to −0.5] ml/min per 1.73 m^2^), as analyzed using a constrained longitudinal data analysis (Figure 3A and Table 3).

**Table 3:** Least squares mean changes from baseline at each visit in GFR and 24-hour urine volume

Changes of Parameters from Baseline	3 mo			6 mo			9 mo			12 mo		
	*n*	LS Mean (SEM)	Differences^a^ (95% CI)	*n*	LS Mean (SEM)	Differences^a^ (95% CI)	*n*	LS Mean (SEM)	Differences^a^ (95% CI)	*n*	LS Mean (SEM)	Differences^a^ (95% CI)
**GFR, ml/min per 1.73 m** ^**2**^												
Theranova	38	−0.2 (0.4)		36	−0.6 (0.4)		36	−0.8 (0.3)		36	−1.0 (0.3)	
High-flux	37	−1.3 (0.5)	−1.1 (−2.4 to 0.2)	35	−2.1 (0.4)	−1.5 (−2.6 to −0.5)^b^	34	−2.3 (0.4)	−1.5 (−2.6 to −0.5)^b^	34	−2.4 (0.4)	−1.4 (−2.4 to −0.5)^b^
**24-h urine volume, ml/d**												
Theranova	38	−114.0 (47.7)		36	−217.0 (56.8)		36	−235.0 (52.3)		36	−230.0 (67.6)	
High-flux	37	−137.0 (59.3)	−23.7 (−173.0 to 125.5)	35	−383.0 (53.3)	−165.8 (−318.0 to −13.2)^b^	34	−418.0 (67.6)	−183.0 (−350.0 to −15.6)^b^	34	−417.0 (85.3)	−187.3 (−401.0 to 25.9)

CI, confidence interval; LS, least squares.

a The differences in GFR and 24-hour urine volume between groups were calculated using a constrained longitudinal data analysis model.

b Group effect is significant (*P* < 0.05).

#### Urine Volume

The LS mean changes (95% CI) of 24-hour urine volume from baseline to 12 months, analyzed using an ANCOVA, were smaller in the Theranova group compared with the high-flux group (Table 2). Similarly, a constrained longitudinal data analysis showed smaller LS mean changes (95% CI) in the Theranova group at 6 and 9 months (6 months: –165.8 [–318.0 to –13.2] ml/d; 9 months: –183.0 [–350.0 to –15.6] ml/d). However, at 12 months, the difference was NS between the groups (Figure 3B and Table 3).

#### Middle Molecules and Inflammatory Cytokines

**Figure 4:**
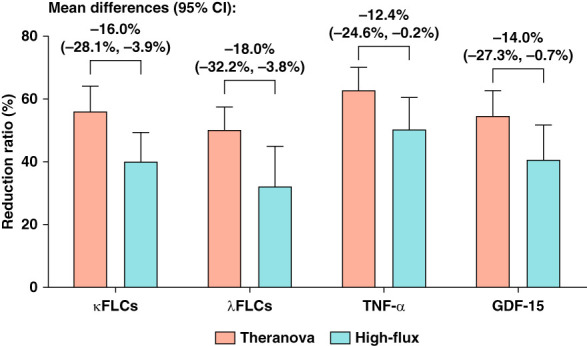
**Reduction ratio of middle molecules and inflammatory cytokines at 12 months.** The bars with lines indicate mean with 95% CI. FLC, free light chain; GDF-15, growth differentiation factor-15.

At 12 months, the Theranova group showed significantly greater removal of middle molecules, such as λ and κ FLCs, and inflammatory cytokines, such as TNF-*α* and GDF-15, compared with the high-flux group. The reduction ratios in the Theranova group were significantly higher, with mean differences (95% CI) as follows: κ FLCs: –16% (–28% to –4%); λ FLCs: –18% (–32% to –4%); TNF-*α*: –12% (–25% to –0.2%); and GDF-15: –14% (–27% to –0.7%) (Figure 4).

When comparing the change in blood levels from baseline to 12 months, *β*2-microglobulin showed a significant decrease in the Theranova group. The LS mean differences in changes (95% CI), determined by an ANCOVA model adjusted for baseline *β*2-microglobulin levels, were 3.9 (1.2 to 6.6) *μ*g/ml (Table 2). However, changes in other variables did not differ significantly between the groups.

#### Markers for Kidney Injury

Markers of kidney injury, such as KIM-1, NGAL, IGFBP7, TIMP-2, and cystatin-C, were comparable at baseline. After 12 months, IGFBP7 levels were significantly lower in the Theranova group, with a mean difference (95% CI) of 12.9 (2.4 to 23.4) ng/ml (Table 2).

When comparing changes from baseline to 12 months, the Theranova group showed significantly smaller increases in IGFBP7 levels compared with the high-flux group. The LS mean difference in changes (95% CI), as determined by an ANCOVA model adjusted for baseline IGFBP7 levels, was 10.0 (–0.7 to 20.7) ng/ml (Table 2).

### Secondary Safety Outcomes

The incidence of hospitalization and mortality over 12 months did not differ between the two groups (Table 2). There was no death in the Theranova group, while two deaths occurred in the high-flux group during the study period, both from cardiovascular disease. None of the deaths were determined to be related to the dialyzer used. There were 12 (30%) adverse events in the Theranova group and 19 (48%) in the high-flux group (Supplemental Table 3). All adverse events were typical of those seen in patients undergoing hemodialysis, and their association with the dialyzer was unclear.

### Exploratory Outcomes

The changes in creatinine clearance and urea clearance, which are components of GFR measured using 24-hour urine collection, were compared between the two groups. The LS mean changes (95% CI) in creatinine clearance from baseline to 6, 9, and 12 months, as analyzed using a constrained longitudinal data analysis model, were consistently lower in the Theranova group compared with the high-flux group (6 months: −2.1 [−3.5 to −0.7] ml/min per 1.73 m^2^; 9 months: −2.3 [−3.7 to −1.0] ml/min per 1.73 m^2^; 12 months: −2.2 [−3.4 to −1.0] ml/min per 1.73 m^2^) (Supplemental Figure 1A and Supplemental Table 4). Similarly, the LS mean changes (95% CI) in urea clearance from baseline to 6 and 9 months, also analyzed using the constrained longitudinal data analysis model, were lower in the Theranova group compared with the high-flux group (6 months: −1.0 [−1.8 to −0.2] ml/min per 1.73 m^2^; 9 months: −0.8 [−1.5 to −0.0] ml/min per 1.73 m^2^) (Supplemental Figure 1B and Supplemental Table 4).

## Discussion

In this randomized controlled trial, expanded hemodialysis with the Theranova dialyzer showed less decline in residual kidney function than the high-flux dialyzer of similar size in patients on newly initiated hemodialysis. The residual kidney function revealed a significant difference between the two groups from 6 months of dialysis and maintained through the 12-month period. The Theranova dialyzer effectively decreased a higher amount of middle molecules and inflammatory cytokines during hemodialysis, and better clearance could contribute to the preservation of residual kidney function in the early stage of dialysis. The Theranova group exhibited a lower alteration in kidney injury markers after 12 months, suggesting a potential protective effect of expanded hemodialysis on native kidney function.

Residual kidney function significantly influences clinical outcomes in patients on dialysis.^9,19^ The potential benefits of residual kidney function may result from improved management of fluid volume, electrolytes, and minerals; reduced inflammation; and more efficient removal of protein-bound waste products and middle molecules.^13^ Even a small amount of residual kidney function helps eliminate uremic toxins and excessive water on nondialysis days, contributing to a better prognosis.^13,20^ Several strategies, such as frequent monitoring of urine volume and residual kidney function, BP control, and avoidance of nephrotoxic events, have been recommended for patients on hemodialysis to maintain residual kidney function.^13,21–23^ Regarding dialysis technologies, biocompatible dialyzers had a better effect on preserving residual kidney function than bioincompatible dialyzers.^24–27^ Some researchers have reported that hemodiafiltration mitigates the decline in residual kidney function compared with hemodialysis with high-flux dialyzers.^28,29^ Although the mechanism remains unclear, high-efficiency biocompatible dialyzers can protect kidney function by reducing inflammation or eliminating inflammatory cytokines.^30^ Expanded hemodialysis with Theranova is also associated with a higher reduction ratio and a lower serum level of TNF-*α*.^8,30^ Theranova significantly reduced C-reactive protein in patients with high levels of baseline C-reactive protein.^31^ Given this context, we conducted a study to analyze the effect of Theranova on residual kidney function in patients initiating dialysis. Our findings proposed that removing large middle molecules could have a beneficial effect on residual kidney function.

In this study, residual kidney function was assessed by GFR. GFR is the leading surrogate marker of kidney function, and 24-hour urine GFR is the primary method for evaluating residual kidney function in patients on dialysis. The Theranova group showed significantly less decline in GFR starting at 6 months of dialysis and maintained the difference through 12 months. Previous studies have investigated the rate of GFR decline after dialysis initiation. In the Netherlands Cooperative Study on the Adequacy of Dialysis, the average decline was −4 ml/min per 1.73 m^2^ over 12 months.^21^ By contrast, the recently published Bio-impedance Spectroscopy to maintain Renal Output study, which applied protocolized hemodialysis, observed a smaller decline of –2.2 ml/min per 1.73 m^2^.^32^ In our study, all patients received the same hemodialysis protocol, and the rate of GFR decline over 12 months was –3.6 ml/min per 1.73 m^2^ in the high-flux group, which was similar to that observed in previous studies. However, in the Theranova group, the rate of GFR decline was –1.4 ml/min per 1.73 m^2^, which was slower than that reported in previous studies.

Although differences in the rate of GFR decline were observed, there was no difference in the absolute GFR values during the study period. This may be attributed to the very low pooled baseline GFR (4.0 ml/min per 1.73 m^2^) and its further decrease over 12 months, which likely limited the detection of significant differences. Similar findings have been reported in other studies evaluating GFR decline in patients initiating dialysis.^32–34^ As a result, many studies on residual kidney function preservation in patients with dialysis have focused on the slope of GFR decline rather than absolute GFR values.

A composite urea and creatinine clearance is used to estimate GFR because urea clearance underestimates GFR compared with creatinine clearance.^35^ Our findings showed that creatinine clearance exhibited consistent changes from 6 months onward, while the significance of urea clearance decreased over time. In a study monitoring GFR among patients on dialysis, the decline in urea clearance was slower than that of creatinine clearance.^36^ Therefore, urea clearance may be an inadequate marker for detecting meaningful alterations within a limited period.

In the maintenance of residual kidney function, the accumulation of uremic toxins can worsen residual kidney function, and their removal is critical for preserving residual kidney function.^37–39^ Several middle-molecular uremic toxins can impair kidney function through nephron loss and interstitial fibrosis.^38,39^ Various studies have shown that the Theranova dialyzer consistently enhanced the removal of middle-molecular uremic toxins without decreasing albumin.^1,8,40^ A recent study revealed that the removal of middle molecules by expanded hemodialysis ameliorated systemic microinflammation and endothelial dysfunction associated with loss of residual kidney function.^41^ In this study, the use of Theranova effectively removed large uremic toxins and proinflammatory substances with a MW above 15 kDa, such as κ/λ FLCs, TNF-*α*, and GDF-15, suggesting its beneficial effect on the preservation of residual kidney function.

However, despite an improved removal rate of middle-MW substances, there were no significant differences in their absolute values or changes during the first 12 months of hemodialysis. This finding is also consistent with previous studies using the Theranova dialyzer, suggesting that although middle-MW substances are removed by dialysis, the effect on newly generated substances is limited.^1^ Moreover, the kinetics of removal, generation, and intercompartmental distribution have not been analyzed and require further investigation for a clear understanding of the mechanism.

In this study, whether the removal of middle molecules by the Theranova might influence not only residual kidney function but also kidney injury markers was investigated. Over 12 months, the degree of elevation of IGFBP7, a marker of kidney tubular injury, seemed smaller in the Theranova group. However, other kidney injury markers, such as KIM-1, NGAL, TIMP-2, and cystatin-C, did not show noticeable differences between the groups. Generally, kidney injury biomarkers are used for evaluating AKI, and the patterns of change can vary depending on the phenotypes of kidney injury.^42^ Therefore, the clinical implication of these markers in patients with kidney failure with various etiologies is not easy to interpret. Nevertheless, the observed changes in some kidney injury markers warrant further investigation to better understand the potential effects of Theranova on residual kidney function.

A recent study has revealed that high-dose hemodiafiltration improves mortality compared with conventional high-flux hemodialysis.^43^ The result suggests that enhanced removal of uremic toxins can lead to improved mortality outcomes in patients on maintenance hemodialysis. Given that expanded hemodialysis preserves residual kidney function by eliminating large middle molecules, further investigation is required to determine whether expanded hemodialysis is associated with better survival. The ongoing large study may demonstrate the efficacy of expanded hemodialysis in comparison with hemodiafiltration on patient survival.^44^

The strengths of the study include its randomized study design, which suggests a potential benefit of the Theranova dialyzer in preserving residual kidney function compared with the conventional high-flux dialyzer in patients newly initiated on hemodialysis. The study also demonstrated effective removal of various middle molecules and the safety of using the Theranova dialyzer in patients with residual kidney function. However, there are several limitations to this study. First, the patients enrolled in the final analysis met our initial target number of participants, but the 12-month duration of the study did not allow for long-term follow-up of changes in residual kidney function. Second, the study was limited in number of patients and duration of observation to determine how preserving residual kidney function early in hemodialysis affects patient mortality, cardiovascular disease risk, and hospitalization. A large-scale, long-term, randomized controlled trial is required to determine the long-term effects of expanded hemodialysis using the Theranova dialyzer on the preservation of residual kidney function and clinical outcomes. Finally, this study did not restrict the use of medications affecting residual kidney function, such as diuretics or antihypertensives, which were used at the discretion of each physician if criteria were met. However, there was no significant difference in the frequency of diuretics or antihypertensives.

The study indicates that expanded hemodialysis using the Theranova dialyzer may help reduce the decrease in residual kidney function compared with the high-flux dialyzer in patients starting treatment with long-term hemodialysis. The Theranova dialyzer removes a wide range of middle-MW uremic toxins and inflammatory substances, which may contribute to preserving residual kidney function. However, further investigation is needed to confirm the renoprotective effects and potential relationship.

## Supplementary Material

SUPPLEMENTARY MATERIAL

## Data Availability

Partial restrictions to the data and/or materials apply. The data that support the findings of this study are available on request from the corresponding author. The data are not publicly available due to their containing information that could compromise the privacy of research participants.

## Supplemental Material

Supplemental Table 1. Medication of the per-protocol population.

Supplemental Table 2. Dialysis treatment information.

Supplemental Table 3. Adverse events.

Supplemental Table 4. LS mean changes from baseline at each visit in creatinine clearance and urea clearance.

Supplemental Figure 1. LS mean changes from baseline to 12 months for creatinine clearance and urea clearance.

Statistical Analysis Plan
