# Supplementary material for: Expanded Hemodialysis with Theranova Dialyzer and Residual Kidney Function in Patients Starting Long-Term Hemodialysis: A Randomized Controlled Trial
Source: J Am Soc Nephrol. 2025 Mar 4;36(8):1614–25. doi: 10.1681/ASN.0000000655 (PMC12342098; doi:10.1681/ASN.0000000655)
Supplement: SUPPLEMENTARY MATERIAL [file jasn-36-1614-s001.pdf]

ASN American Society of Nephrology
JASN Journal of the American Society of Nephrology
KIDNEY360 Accessing Our World From Every Angle
CJASN Clinical Journal of the American Society of Nephrology

ASN Journal Disclosure Form

As per ASN journal policy, I have disclosed any financial relationships or commitments I have held in the past 36 months as included below. I have listed my Current Employer below to indicate there is a relationship requiring disclosure. If no relationship exists, my Current Employer is not listed.

J. Cho reports the following:
Employer: Kyungpook National University Hospital; and Research Funding: Baxter.

I understand that the information above will be published within the journal article, if accepted, and that failure to comply and/or to accurately and completely report the potential financial conflicts of interest could lead to the following: 1) Prior to publication, article rejection, or 2) Post-publication, sanctions ranging from, but not limited to, issuing a correction, reporting the inaccurate information to the authors' institution, banning authors from submitting work to ASN journals for varying lengths of time, and/or retraction of the published work.

Name: Jang-Hee Cho
Manuscript ID: JASN-2024-001148R3
Manuscript Title: Expanded Hemodialysis with Theranova Dialyzer and Residual Kidney Function in Incident Hemodialysis Patients: A Randomized Controlled Trial
Date of Completion: February 13, 2025
Disclosure Updated Date: January 31, 2025

ASN American Society of Nephrology
JASN Journal of the American Society of Nephrology
KIDNEY360 Accessing Our World From Every Angle
CJASN Clinical Journal of the American Society of Nephrology

ASN Journal Disclosure Form

As per ASN journal policy, I have disclosed any financial relationships or commitments I have held in the past 36 months as included below. I have listed my Current Employer below to indicate there is a relationship requiring disclosure. If no relationship exists, my Current Employer is not listed.

J. Choi reports the following:
Employer: Kyungpook National University Chilgok Hospital

I understand that the information above will be published within the journal article, if accepted, and that failure to comply and/or to accurately and completely report the potential financial conflicts of interest could lead to the following: 1) Prior to publication, article rejection, or 2) Post-publication, sanctions ranging from, but not limited to, issuing a correction, reporting the inaccurate information to the authors' institution, banning authors from submitting work to ASN journals for varying lengths of time, and/or retraction of the published work.

Name: Ji-Young Choi
Manuscript ID: JASN-2024-001148R3
Manuscript Title: Expanded Hemodialysis with Theranova Dialyzer and Residual Kidney Function in Incident Hemodialysis Patients: A Randomized Controlled Trial
Date of Completion: February 19, 2025
Disclosure Updated Date: February 19, 2025

ASN American Society of Nephrology
JASN Journal of the American Society of Nephrology
KIDNEY360 Accessing Our World From Every Angle
CJASN Clinical Journal of the American Society of Nephrology

ASN Journal Disclosure Form

As per ASN journal policy, I have disclosed any financial relationships or commitments I have held in the past 36 months as included below. I have listed my Current Employer below to indicate there is a relationship requiring disclosure. If no relationship exists, my Current Employer is not listed.

Y. Jeon has nothing to disclose.

I understand that the information above will be published within the journal article, if accepted, and that failure to comply and/or to accurately and completely report the potential financial conflicts of interest could lead to the following: 1) Prior to publication, article rejection, or 2) Post-publication, sanctions ranging from, but not limited to, issuing a correction, reporting the inaccurate information to the authors' institution, banning authors from submitting work to ASN journals for varying lengths of time, and/or retraction of the published work.

Name: Yena Jeon
Manuscript ID: JASN-2024-001148R3
Manuscript Title: Expanded Hemodialysis with Theranova Dialyzer and Residual Kidney Function in Incident Hemodialysis Patients: A Randomized Controlled Trial.
Date of Completion: February 19, 2025
Disclosure Updated Date: February 19, 2025

ASN American Society of Nephrology
JASN Journal of the American Society of Nephrology
KIDNEY360 Accessing Our World From Every Angle
CJASN Clinical Journal of the American Society of Nephrology

ASN Journal Disclosure Form

As per ASN journal policy, I have disclosed any financial relationships or commitments I have held in the past 36 months as included below. I have listed my Current Employer below to indicate there is a relationship requiring disclosure. If no relationship exists, my Current Employer is not listed.

Y. Jeon reports the following:
Employer: Kyungpook National University Hospital

I understand that the information above will be published within the journal article, if accepted, and that failure to comply and/or to accurately and completely report the potential financial conflicts of interest could lead to the following: 1) Prior to publication, article rejection, or 2) Post-publication, sanctions ranging from, but not limited to, issuing a correction, reporting the inaccurate information to the authors' institution, banning authors from submitting work to ASN journals for varying lengths of time, and/or retraction of the published work.

Name: You Hyun Jeon
Manuscript ID: JASN-2024-001148R1
Manuscript Title: Expanded Hemodialysis with Theranova Dialyzer and Residual Kidney Function in Incident Hemodialysis Patients: A Randomized Controlled Trial
Date of Completion: December 19, 2024
Disclosure Updated Date: December 19, 2024

ASN American Society of Nephrology | JASN Journal of the American Society of Nephrology | KIDNEY360 Accessing Our World From Every Angle | CJASN Clinical Journal of the American Society of Nephrology

ASN Journal Disclosure Form

As per ASN journal policy, I have disclosed any financial relationships or commitments I have held in the past 36 months as included below. I have listed my Current Employer below to indicate there is a relationship requiring disclosure. If no relationship exists, my Current Employer is not listed.

H. Jung has nothing to disclose.

I understand that the information above will be published within the journal article, if accepted, and that failure to comply and/or to accurately and completely report the potential financial conflicts of interest could lead to the following: 1) Prior to publication, article rejection, or 2) Post-publication, sanctions ranging from, but not limited to, issuing a correction, reporting the inaccurate information to the authors' institution, banning authors from submitting work to ASN journals for varying lengths of time, and/or retraction of the published work.

Name: Hee-Yeon Jung
Manuscript ID: JASN-2024-001148R3
Manuscript Title: Expanded Hemodialysis with Theranova Dialyzer and Residual Kidney Function in Incident Hemodialysis Patients: A Randomized Controlled Trial
Date of Completion: February 14, 2025
Disclosure Updated Date: May 14, 2024

ASN American Society of Nephrology
JASN Journal of the American Society of Nephrology
KIDNEY360 Accessing Our World From Every Angle
CJASN Clinical Journal of the American Society of Nephrology

ASN Journal Disclosure Form

As per ASN journal policy, I have disclosed any financial relationships or commitments I have held in the past 36 months as included below. I have listed my Current Employer below to indicate there is a relationship requiring disclosure. If no relationship exists, my Current Employer is not listed.

D. Kang reports the following:
Employer: Ewha University College of Medicine; Seoul, Korea

I understand that the information above will be published within the journal article, if accepted, and that failure to comply and/or to accurately and completely report the potential financial conflicts of interest could lead to the following: 1) Prior to publication, article rejection, or 2) Post-publication, sanctions ranging from, but not limited to, issuing a correction, reporting the inaccurate information to the authors' institution, banning authors from submitting work to ASN journals for varying lengths of time, and/or retraction of the published work.

Name: Duk-Hee Kang
Manuscript ID: JASN-2024-001148R3
Manuscript Title: Expanded Hemodialysis with Theranova Dialyzer and Residual Kidney Function in Incident Hemodialysis Patients: A Randomized Controlled Trial
Date of Completion: February 23, 2025
Disclosure Updated Date: May 22, 2024

ASN
American Society of Nephrology
JASN
Journal of the American Society of Nephrology
KIDNEY360
Accessing Our World From Every Angle
CJASN
Clinical Journal of the American Society of Nephrology

ASN Journal Disclosure Form

As per ASN journal policy, I have disclosed any financial relationships or commitments I have held in the past 36 months as included below. I have listed my Current Employer below to indicate there is a relationship requiring disclosure. If no relationship exists, my Current Employer is not listed.

S. Kang has nothing to disclose.

I understand that the information above will be published within the journal article, if accepted, and that failure to comply and/or to accurately and completely report the potential financial conflicts of interest could lead to the following: 1) Prior to publication, article rejection, or 2) Post-publication, sanctions ranging from, but not limited to, issuing a correction, reporting the inaccurate information to the authors' institution, banning authors from submitting work to ASN journals for varying lengths of time, and/or retraction of the published work.

Name: Seok hui Kang
Manuscript ID: JASN-2024-001148R3
Manuscript Title: Expanded Hemodialysis with Theranova Dialyzer and Residual Kidney Function in Incident Hemodialysis Patients: A Randomized Controlled Trial
Date of Completion: February 19, 2025
Disclosure Updated Date: February 19, 2025

ASN
American Society of Nephrology
JASN
Journal of the American Society of Nephrology
KIDNEY360
Accessing Our World From Every Angle
CJASN
Clinical Journal of the American Society of Nephrology

ASN Journal Disclosure Form

As per ASN journal policy, I have disclosed any financial relationships or commitments I have held in the past 36 months as included below. I have listed my Current Employer below to indicate there is a relationship requiring disclosure. If no relationship exists, my Current Employer is not listed.

C. Kim reports the following:
Employer: Kyungpook University Hospital

I understand that the information above will be published within the journal article, if accepted, and that failure to comply and/or to accurately and completely report the potential financial conflicts of interest could lead to the following: 1) Prior to publication, article rejection, or 2) Post-publication, sanctions ranging from, but not limited to, issuing a correction, reporting the inaccurate information to the authors' institution, banning authors from submitting work to ASN journals for varying lengths of time, and/or retraction of the published work.

Name: Chan-Duck Kim
Manuscript ID: JASN-2024-001148R3
Manuscript Title: Expanded Hemodialysis with Theranova Dialyzer and Residual Kidney Function in Incident Hemodialysis Patients: A Randomized Controlled Trial
Date of Completion: February 19, 2025
Disclosure Updated Date: February 19, 2025

ASN American Society of Nephrology | JASN Journal of the American Society of Nephrology | KIDNEY360 Accessing Our World From Every Angle | CJASN Clinical Journal of the American Society of Nephrology

ASN Journal Disclosure Form

As per ASN journal policy, I have disclosed any financial relationships or commitments I have held in the past 36 months as included below. I have listed my Current Employer below to indicate there is a relationship requiring disclosure. If no relationship exists, my Current Employer is not listed.

Y. Kim reports the following:
Employer: Kyungpook National University; Research Funding: Baxter; Honoraria: Baxter; Advisory or Leadership Role: [Journal]Editorial Board, Ther Apher Dialysis; [Journal]Editorial Board, Life; [Journal]Editorial Board, Perit Dial Int; [Journal] Editorial Board, Scientific Reports; and Other Interests or Relationships: American Society of Nephrology; Korean Society of Nephrology; ISPD; ISN; ISFA.

I understand that the information above will be published within the journal article, if accepted, and that failure to comply and/or to accurately and completely report the potential financial conflicts of interest could lead to the following: 1) Prior to publication, article rejection, or 2) Post-publication, sanctions ranging from, but not limited to, issuing a correction, reporting the inaccurate information to the authors' institution, banning authors from submitting work to ASN journals for varying lengths of time, and/or retraction of the published work.

Name: Yong-Lim Kim
Manuscript ID: JASN-2024-001148R3
Manuscript Title: Expanded Hemodialysis with Theranova Dialyzer and Residual Kidney Function in Incident Hemodialysis Patients: A Randomized Controlled Trial.
Date of Completion: February 13, 2025
Disclosure Updated Date: December 20, 2024

ASN American Society of Nephrology | JASN Journal of the American Society of Nephrology | KIDNEY360 Accessing Our World From Every Angle | CJASN Clinical Journal of the American Society of Nephrology

ASN Journal Disclosure Form

As per ASN journal policy, I have disclosed any financial relationships or commitments I have held in the past 36 months as included below. I have listed my Current Employer below to indicate there is a relationship requiring disclosure. If no relationship exists, my Current Employer is not listed.

J. Lim reports the following:
Employer: Kyungpook National University Hospital

I understand that the information above will be published within the journal article, if accepted, and that failure to comply and/or to accurately and completely report the potential financial conflicts of interest could lead to the following: 1) Prior to publication, article rejection, or 2) Post-publication, sanctions ranging from, but not limited to, issuing a correction, reporting the inaccurate information to the authors' institution, banning authors from submitting work to ASN journals for varying lengths of time, and/or retraction of the published work.

Name: Jeong-Hoon Lim
Manuscript ID: JASN-2024-001148R1
Manuscript Title: Expanded Hemodialysis with Theranova Dialyzer and Residual Kidney Function in Incident Hemodialysis Patients: A Randomized Controlled Trial
Date of Completion: December 19, 2024
Disclosure Updated Date: December 19, 2024

ASN American Society of Nephrology JASN Journal of the American Society of Nephrology KIDNEY360 Accessing Our World From Every Angle CJASN Clinical Journal of the American Society of Nephrology

ASN Journal Disclosure Form

As per ASN journal policy, I have disclosed any financial relationships or commitments I have held in the past 36 months as included below. I have listed my Current Employer below to indicate there is a relationship requiring disclosure. If no relationship exists, my Current Employer is not listed.

S. Park reports the following:
Employer: Kyungpook National University Hospital

I understand that the information above will be published within the journal article, if accepted, and that failure to comply and/or to accurately and completely report the potential financial conflicts of interest could lead to the following: 1) Prior to publication, article rejection, or 2) Post-publication, sanctions ranging from, but not limited to, issuing a correction, reporting the inaccurate information to the authors' institution, banning authors from submitting work to ASN journals for varying lengths of time, and/or retraction of the published work.

Name: Sun-Hee Park
Manuscript ID: JASN-2024-001148R2
Manuscript Title: Expanded Hemodialysis with Theranova Dialyzer and Residual Kidney Function in Incident Hemodialysis Patients: A Randomized Controlled Trial
Date of Completion: February 2, 2025
Disclosure Updated Date: February 2, 2025

ASN Journal Disclosure Form

As per ASN journal policy, I have disclosed any financial relationships or commitments I have held in the past 36 months as included below. I have listed my Current Employer below to indicate there is a relationship requiring disclosure. If no relationship exists, my Current Employer is not listed.

J. Ryu reports the following:
Employer: Ewha Womans University College of Medicine

I understand that the information above will be published within the journal article, if accepted, and that failure to comply and/or to accurately and completely report the potential financial conflicts of interest could lead to the following: 1) Prior to publication, article rejection, or 2) Post-publication, sanctions ranging from, but not limited to, issuing a correction, reporting the inaccurate information to the authors' institution, banning authors from submitting work to ASN journals for varying lengths of time, and/or retraction of the published work.

Name: Jung-hwa Ryu
Manuscript ID: JASN-2024-001148R1
Manuscript Title: Expanded Hemodialysis with Theranova Dialyzer and Residual Kidney Function in Incident Hemodialysis Patients: A Randomized Controlled Trial
Date of Completion: December 19, 2024
Disclosure Updated Date: December 19, 2024

ASN American Society of Nephrology JASN Journal of the American Society of Nephrology KIDNEY360 Accessing Our World From Every Angle CJASN Clinical Journal of the American Society of Nephrology

ASN Journal Disclosure Form

As per ASN journal policy, I have disclosed any financial relationships or commitments I have held in the past 36 months as included below. I have listed my Current Employer below to indicate there is a relationship requiring disclosure. If no relationship exists, my Current Employer is not listed.

Y. Seo has nothing to disclose.

I understand that the information above will be published within the journal article, if accepted, and that failure to comply and/or to accurately and completely report the potential financial conflicts of interest could lead to the following: 1) Prior to publication, article rejection, or 2) Post-publication, sanctions ranging from, but not limited to, issuing a correction, reporting the inaccurate information to the authors' institution, banning authors from submitting work to ASN journals for varying lengths of time, and/or retraction of the published work.

Name: YU JIN Seo
Manuscript ID: JASN-2024-001148R3
Manuscript Title: Expanded Hemodialysis with Theranova Dialyzer and Residual Kidney Function in Incident Hemodialysis Patients: A Randomized Controlled Trial
Date of Completion: February 18, 2025
Disclosure Updated Date: February 18, 2025
