# Supplementary material for: Expanded Hemodialysis with Theranova Dialyzer and Residual Kidney Function in Patients Starting Long-Term Hemodialysis: A Randomized Controlled Trial
Source: J Am Soc Nephrol. 2025 Mar 4;36(8):1614–25. doi: 10.1681/ASN.0000000655 (PMC12342098; doi:10.1681/ASN.0000000655)
Supplement: SUPPLEMENTARY MATERIAL [file jasn-36-1614-s002.pdf]

Expanded Hemodialysis with Theranova Dialyzer and Residual Kidney Function in Incident Hemodialysis Patients: A Randomized Controlled Trial

Supplemental Material Table of Contents

Supplemental Table 1. Medication of the per-protocol population

Supplemental Table 2. Dialysis treatment information

Supplemental Table 3. Adverse events

Supplemental Table 4. Least-square mean changes from baseline at each visit in creatinine clearance and urea clearance

Supplemental Figure 1. Least-squares mean changes from baseline to 12 months for creatinine clearance and urea clearance.

Statistical Analysis Plan

Supplemental Table 1. Medication of the per-protocol population

	Theranova (*n* = 36)	High-flux (*n* = 34)
RAAS inhibitor, *n* (%)	29 (81)	30 (88)
Beta blocker, *n* (%)	21 (58)	23 (68)
Calcium channel blocker, *n* (%)	33 (92)	28 (82)
Alpha blocker, *n* (%)	8 (22)	6 (18)
Minoxidil, *n* (%)	1 (3)	2 (6)
Loop diuretics, *n* (%)	24 (67)	22 (65)
Thiazide, *n* (%)	3 (8)	4 (12)
Aldosterone antagonist, *n* (%)	0	1 (3)

Abbreviation: RAAS, Renin-angiotensin-aldosterone system.

Supplemental Table 2. Dialysis treatment information

Parameter	Visit months	Theranova *n* mean (SD)	High-flux *n* mean (SD)	Differences *n* mean (95% CI)
Blood flow rate, mL/min	0	40<br>194.0 (17.4)	40<br>194.3 (18.5)	40<br>0.3 (–7.6, 8.1)
	6	36<br>217.2 (23.3)	35<br>219.1 (33.6)	35<br>1.9 (–11.5, 15.4)
	12	36<br>225.0 (32.1)	34<br>229.1 (30.7)	34<br>4.1 (–10.6, 18.8)
Dialysate flow rate, mL/min	0	40<br>522.5 (42.3)	40<br>515.0 (36.2)	40<br>–7.5 (–24.7, 9.7)
	6	36<br>522.2 (42.2)	35<br>511.4 (32.3)	35<br>–10.8 (–28.2, 6.6)
	12	36<br>522.2 (42.2)	34<br>511.8 (32.7)	34<br>–10.5 (–28.1, 7.2)
Ultrafiltration volume, L	0	40<br>1.3 (1.0)	40<br>1.2 (0.8)	40<br>–0.1 (–0.5, 0.3)
	6	36<br>1.7 (0.9)	35<br>1.7 (0.9)	35<br>–0.0 (–0.4, 0.4)
	12	36<br>2.1 (0.9)	34<br>1.8 (1.0)	34<br>–0.2 (–0.7, 0.2)
Treatment time, h	0	40<br>4.0 (0)	40<br>3.9 (0.1)	40<br>–0.0 (–0.0, 0.0)
	6	36<br>4.0 (0)	35<br>4.0 (0)	35<br>0.0 (0.0, 0.0)
	12	36<br>4.0 (0)	34<br>4.0 (0)	34<br>0.0 (0.0, 0.0)

Abbreviation: CI, confidence interval.

Supplemental Table 3. Adverse events

	Theranova (*n* = 40)	High-flux (*n* = 40)
Chest discomfort, *n* (%)	1 (3)	1 (3)
Dyspnea, *n* (%)	1 (3)	2 (5)
Atrial flutter, *n* (%)	0	1 (3)
Seizure, *n* (%)	0	1 (3)
Arteriovenous fistula/graft thrombosis, *n* (%)	3 (8)	2 (5)
Diabetic foot ulcer, *n* (%)	1 (3)	1 (3)
Major depressive disorder, *n* (%)	1 (3)	0
Anal fistula, *n* (%)	1 (3)	0
Herpes zoster, *n* (%)	1 (3)	0
Enterocolitis, *n* (%)	0	1 (3)
Cecal cancer, *n* (%)	0	1 (3)
Pneumonia, *n* (%)	0	1 (3)
Urinary tract infection, *n* (%)	1 (3)	0
Insomnia, *n* (%)	1 (3)	4 (10)

Supplemental Table 4. Least-squares mean changes from baseline at each visit in creatinine clearance and urea clearance

Changes of parameters from baseline	3 months			6 months			9 months			12 months		
	n	LS mean (SE)	Differences[a] (95% CI)	*n*	LS mean (SE)	Differences[a] (95% CI)	*n*	LS mean (SE)	Differences[a] (95% CI)	*n*	LS mean (SE)	Differences[a] (95% CI)
Creatinine clearance, mL/min/1.73 m^2												
Theranova	38	–0.4 (0.5)		36	–0.8 (0.5)		36	–1.1 (0.4)		36	–1.4 (0.4)	
High-flux	37	–2.0 (0.7)	–1.6 (–3.3, 0.2)	35	–2.9 (0.5)	–2.1 (–3.5, –0.7)†	34	–3.4 (0.6)	–2.3 (–3.7, –1.0)†	34	–3.6 (0.5)	–2.2 (–3.4, –1.0)†
Urea clearance, mL/min/1.73 m^2												
Theranova	38	–0.01 (0.4)		36	–0.3 (0.3)		36	–0.5 (0.3)		36	–0.5 (0.3)	
High-flux	37	–0.6 (0.3)	–0.6 (–1.5, 0.4)	35	–1.2 (0.2)	–1.0 (–1.8, –0.2)†	34	–1.2 (0.3)	–0.8 (–1.5, –0.0)†	34	–1.2 (0.3)	–0.7 (–1.5, 0.1)

[a]The differences in creatinine clearance and urea clearance between groups were calculated using a constrained longitudinal data analysis model.

†Differences between groups are significant.

Abbreviations: LS, least-squares; SE, standard error; CI, confidence interval.

Supplemental Figure 1. Least-squares mean changes from baseline to 12 months for creatinine clearance (A) and urea clearance (B).

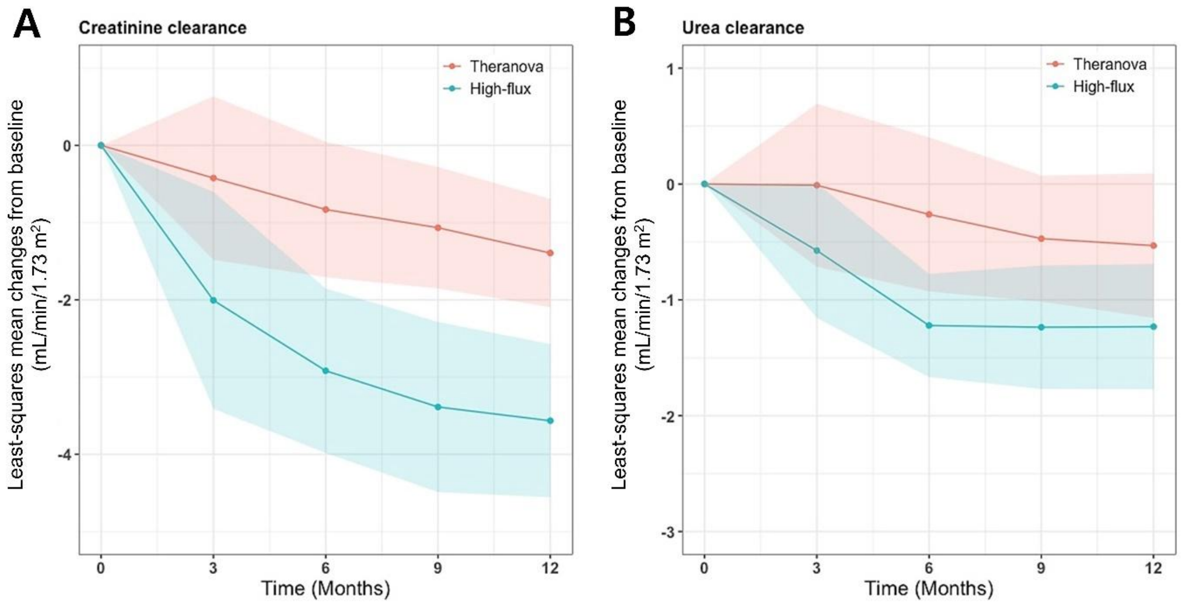

Data are presented as least-squares mean changes with 95% confidence intervals, which were calculated using a constrained longitudinal data analysis model.

Statistical Analysis Plan

Expanded Hemodialysis with Theranova Dialyzer Preserves Residual Kidney Function in Incident Hemodialysis Patients: The THREAD Randomized Controlled Trial

Principal Investigator:	Yong-Lim Kim, MD, PhD<br>Professor of Internal Medicine<br>Kyungpook National University Hospital<br>Daegu, Republic of Korea
ClinicalTrials.gov identifier:	04211571
Trial Statistician:	Yu Jin Seo, PhD<br>Department of Statistics<br>Kyungpook National University<br>Daegu, Republic of Korea
Protocol Version and Date:	Version 1.0 (dated 15-01-2020)

Signature Page

Principal Investigator: ______________________

Yong-Lim Kim (15-01-2020)

Trial Statistician: 서 유진

Yu Jin Seo (15-01-2020)

Table of contents

1. Introduction.. 5

1.1 Background... 5

1.2 Aims and objectives.. 5

2. Study methods... 5

2.1 Trial design.. 5

2.2 Randomization... 6

2.3 Sample size calculation... 6

2.4 Timing of final analysis.. 7

3. Outcomes... 7

3.1 Primary outcome.. 7

3.2 Secondary outcomes 7

3.3 Safety outcomes.. 8

4. Trial population.. 8

5. Analyses.. 9

5.1 Primary outcome.. 9

5.2 Secondary outcomes 9

5.3 Safety outcomes 10

6. Missing data 10

7. References 11

1. Introduction

1.1 Background

Expanded hemodialysis (HDx) using a medium cut-off (MCO) dialyzer can improve the clearance of middle-molecular uremic toxins compared to conventional hemodialysis (HD). Several studies have validated a superior efficacy of HDx over conventional HD, demonstrating clinical advantages, such as ameliorating quality of life, pruritus, and anemia. However, the effect of HDx on residual kidney function (RKF) remains unclear. This multicenter randomized controlled clinical trial aims to evaluate the effect of HDx using Theranova dialyzer on preserving RKF in incident HD patients.

This statistical analysis plan (SAP) will provide more detailed descriptions of the endpoints in the study and the corresponding analyses.

1.2 Aims and objectives

This study aims to determine whether the use of Theranova, a high-efficiency membrane, helps preserve RKF compared to conventional high-flux dialysis dialyzer in patients newly initiated on hemodialysis after a diagnosis of end-stage kidney disease (ESKD). In addition, we will provide a theoretical basis for the RKF preservation effect by measuring changes in middle molecules known to affect RKF, and confirm the effect of improving quality of life through RKF preservation.

2. Study methods

2.1 Trial design

This is a multicenter, open-label, prospective, parallel-group study in which patients with newly diagnosed ESKD and initiated hemodialysis. Study subjects will be recruited from 4 tertiary hospitals in the Republic of Korea and will be randomized 1:1 to receive Theranova, an MCO membrane, or a control group using a conventional high-flux dialyzer (Fx CorDiax 80) membrane. To determine the RKF preservation effect of the Theranova dialyzer, patients will perform hemodialysis for 12 months after randomization and changes in RKF will be compared. RKF is assessed by glomerular filtration rate as measured by 24-hour urine collection. Patients will also be evaluated for changes in residual urine volume and removal of middle molecular uremic toxins, changes in kidney injury markers, and changes in quality of life questionnaire. Enrolled patients will have a total of 5 study visits (0, 3, 6, 9, 12 months).

2.2 Randomization

Patients who meet the inclusion/exclusion criteria as a result of the screening will be randomized in a 1:1 ratio to the Theranova group and the high-flux group. Randomization will be performed using a random number table method by a statistician unrelated to the study. The statistician will assign a randomization code to the Theranova and high-flux groups, assign a registration number, and inform the institution of the randomization results.

2.3 Sample size calculation

The primary outcome measure for the power calculation is the difference between Theranova and high-flux groups in the changes in RKF between baseline and 12 months. There are no previous studies on the effect of the Theranova dialyzer on RKF. Therefore, we determined the sample size based on a study that compared RKF by dialysis fluid quality. The mean glomerular filtration rate (GFR) measured by 24-hour urine collection was 4.3 ± 1.8 mL/min at 12 months in patients newly initiated on HD, with a statistically significant difference of more than 1.7

mL/min [Schiffl et al. *Nephrol Dial Transplant*. 2002;17(10):1814-1818. doi:10.1093/ndt/17.10.1814]. Given this, assuming 80% power, a two-sided type 1 error rate of 5%, and a standard deviation of the RKF difference of 2.5 mL/min, the number of subjects needed was 34 in each arm. We also predicted a 15% dropout rate for both groups; thus, a total sample size of 80 was calculated.

2.4 Timing of final analysis

The trial will finish with the last 12-month follow-up appointment (scheduled around the end of December 2023). The data will be cleaned, verified, and locked. Final analysis will commence once the Principal Investigator has confirmed the final lock.

3. Outcomes

This section will present the outcomes investigated to answer the study aims and objectives. The analyses are described in section 5. Analyses.

3.1 Primary outcome

Change in RKF between baseline and 12 months, which is estimated by GFR using 24-hour urine collection. GFR was calculated as the mean of 24-hour urine creatinine and urea clearance, corrected for a body surface area of 1.73 m^2.

3.2 Secondary outcomes

1) Change in RKF and urine volume every 3 months, change in urine creatinine and urea clearance, the component of RKF every 3 months.

2) The reduction ratio of middle molecules, such as κ and λ free light chains, and inflammatory

cytokines, such as tumor necrosis factor-alpha (TNF-α) and growth differentiation factor-15 (GDF-15), at 12 months

3) The change between baseline and 12 months in middle molecules (κ and λ free light chains, and β2-microglobulin), inflammation-related markers (TNF-α, GDF-15, and high-sensitivity C-reactive protein), and kidney injury markers (neutrophil gelatinase-associated lipocalin NGAL], kidney injury molecule-1 [KIM-1], insulin-like growth factor binding protein 7 [IGFBP7], tissue inhibitor of metalloproteinases-2 [TIMP-2], and cystatin-C).

4) Patient-reported quality of life at 12 months using the Kidney Disease Quality of Life (KDQOL) Short Form version 1.3.

3.3 Safety outcomes

Adverse events are reported at each visit and usage of medications during the study period will be recorded. Serum albumin levels between baseline and 12 months in the Theranova and high-flux groups will be compared. The incidence of hospitalization and mortality will also be recorded for the safety outcomes.

4. Trial population

Key demographic and other baseline characteristics that may influence study outcomes will be summarized per randomized group and overall, as appropriate given the nature of the variable.

Intention-to-treat (ITT): All randomized study subjects. This will be seen as the primary population for the analysis.

Per Protocol (PP): All randomized study subjects completing the whole study period (complete

cases). For a specific analysis, study subjects with missing data on any of the variables in the model will be excluded from the analysis. Analyses of this population is seen as a sensitivity analysis to investigate whether conclusions are sensitive to assumptions regarding the pattern of missing data.

5. Analyses

All outcomes will be presented using descriptive statistics; normally distributed data by the mean and standard deviation (SD) and skewed distributions by the median and interquartile range (IQR). The Shapiro-Wilk test was used to analyze the distribution normality of measured variables. Binary and categorical variables will be presented using numbers and percentages. Statistical analyses will be conducted using SPSS version 22.0 (IBM Corp., Armonk, NY, USA) and R (R Foundation for Statistical Computing, Vienna, Austria; www.r-project.org). The subsections below will describe analyses in addition to the descriptive statistics.

5.1 Primary outcome

The primary analysis will compare changes in GFR between baseline and 12 months in the Theranova and high-flux groups using the Student's t-test or Mann-Whitney U test, depending on the nature and distribution. Differences in GFR measured by 24-hour urine collection from baseline to 12 months will be the dependent variable.

5.2 Secondary outcomes

1) The changes in GFR from baseline to each follow-up period (3, 6, and 9 months) will be compared using the Student's t-test or Mann-Whitney U test. Serial changes at 3-month intervals in creatinine and urea clearance will be analyzed using the same method for the

changes in GFR. The alterations in 24-hour urine volume from baseline to time points (3, 6, 9, and 12 months) will be evaluated utilizing a consistent analytical approach.

2) The reduction ratios of middle molecules will be quantified by measuring their concentration before and after dialysis at 12 months. Depending on the nature and distribution of the data, the reduction ratios will be analyzed using either the Student's t-test or the Mann-Whitney U test.

3) The changes between baseline and 12 months in middle molecules, inflammation-related markers, and kidney injury markers will be compared using either the Student's t-test or Mann-Whitney U test, depending on the nature and distribution.

4) The quality of life will be compared using the mean or median scores for each item of the KDQOL Short Form version 1.3 questionnaire at 12 months by the Student's t-test or Mann-Whitney U test, depending on the nature and distribution of the data.

5.3 Safety outcomes

The incidence of all adverse events reported during the study period will be compared using Pearson's chi-square or Fisher's exact test for both groups. Serum albumin levels between baseline and 12 months in the Theranova and high-flux groups will be analyzed using the Student's t-test or Mann-Whitney U test. The incidence of hospitalization and mortality will be analyzed using Pearson's chi-square test or Fisher's exact test.

6. Missing data

Participants with missing values will be excluded from the analysis of that variable exclusively;

they will not be excluded from all analyses.

7. References

1) Boschetti-de-Fierro A, Voigt M, Storr M, et al. MCO Membranes: Enhanced Selectivity in High-Flux Class. Sci Rep 2015; 5: 18448.

2) Ronco C. The Rise of Expanded Hemodialysis. Blood Purif 2017; 44: I-viii.

3) Schepers E, Glorieux G, Eloot S, et al. Assessment of the association between increasing membrane pore size and endotoxin permeability using a novel experimental dialysis simulation set-up. BMC Nephrol 2018; 19: 1.

4) Kirsch AH, Lyko R, Nilsson LG, et al. Performance of hemodialysis with novel medium cut-off dialyzers. Nephrol Dial Transplant 2017; 32: 165-172.

5) Lim JH, Park Y, Yook JM, et al. Randomized controlled trial of medium cut-off versus high-flux dialyzers on quality of life outcomes in maintenance hemodialysis patients. Sci Rep 2020; 10: 7780.

6) Lim JH, Jeon Y, Yook JM, et al. Medium cut-off dialyzer improves erythropoiesis stimulating agent resistance in a hepcidin-independent manner in maintenance hemodialysis patients: results from a randomized controlled trial. Sci Rep 2020; 10: 16062.

7) Schiffl H and Lang SM, and Fischer R. Ultrapure dialysis fluid slows loss of residual renal function in new dialysis patients. Nephrol Dial Transplant. 2002;17(10):1814-1818.
